# Delayed Axillary Artery Occlusion after Reverse Total Shoulder Arthroplasty

**DOI:** 10.1155/2016/5463514

**Published:** 2016-07-31

**Authors:** Omar M. Ghanem, Jana Sacco, Richard F. Heitmiller, Seyed Mojtaba Gashti

**Affiliations:** ^1^Medstar Union Memorial Hospital, Baltimore, MD 21218, USA; ^2^Saba University School of Medicine, Devens, MA 01434, USA

## Abstract

Axillary artery injury has been associated with shoulder dislocation and surgery. We describe a case of delayed axillary artery occlusion after reverse total shoulder arthroplasty. The injury was confirmed by Doppler and angiography and was treated with angioplasty and stenting. Early recognition and treatment of this injury are mandatory for patients' recovery.

## 1. Introduction

The anatomic location of the axillary artery renders it susceptible to injury after shoulder surgery or dislocation [1]. Although uncommon, most of the reported cases of axillary artery injury appear to be secondary to anterior shoulder dislocation [2]. Axillary artery injury has also been rarely described during shoulder arthroplasty [3]. On the other hand, the relatively new reverse total shoulder arthroplasty (RTSA) has been linked to vascular complications of which hematoma and phlebitis seem to be the most common. However, these complications are typically mild and transient [1]. We present a case of delayed axillary artery occlusion after RTSA.

## 2. Case

This is a case of a 60-year-old man with previous history of type 2 diabetes mellitus and peripheral vascular disease who underwent left shoulder conversion of failed hemiarthroplasty to RTSA. The early postoperative period was within the expected course. However, one month postoperatively, the patient developed left hand throbbing, burning pain, and hand stiffness which kept worsening despite motion exercises. At that time, he had palpable radial pulses and good capillary refill and no further vascular studies were obtained. He was started on NSAIDs and began hand physical therapy. Eight weeks postoperatively, the patient's pain persisted and at this time, his pain was believed to be due to nerve compression and/or complex regional pain syndrome. Thus, he was started on pregabalin and methylprednisolone. Ten weeks after the surgery, the patient continued to have left hand pain, numbness, and tingling and his left hand began to feel cold. On physical exam his brachial, ulnar, and radial pulses were not palpable. Upper extremity arterial Doppler was obtained and showed biphasic waveforms with decreased pressures in the left brachial, radial, and ulnar arteries at rest. The left 1st–3rd digit pressures were decreased and the 4th-5th digits were inaudible. The digital brachial index (DBI) for digits one through five was 0.4, 0.32, 0.31, 0.00, and 0.00, respectively, consistent with moderate to severe ischemia. The right brachial, radial, and ulnar waveforms and pressures were essentially normal. Therefore, it was decided to proceed with angiography to better define his ischemic injury.

## 3. Procedures

### 3.1. Reverse Total Shoulder Arthroplasty

The deltopectoral interval was mobilized, releasing the deltoid sleeve distally. Furthermore, circumferential medial and lateral exposure was performed, releasing prior anterior lesser tuberosity. The posterior rotator cuff was incised as it was healed in a posterior-superior position and the greater tuberosity osteotomized with a saw and removed as well. The poor cement mantel was removed along with the stem. The humerus was recut in the proper neck shaft angle and the canal was curetted removing any prior slime material and debris. It was reamed up to an 11 mm reamer and broached to an 11 mm stem. Afterwards, circumferential exposure of the glenoid was performed and the bicipital tendon was tenotomized and excised, completing the biceps tendon resection. The glenoid was prepared in standard fashion for a standard base plate, which was secured with a central screw and 4 nonlocking screws with good compression. The +3 × 41 mm Glenosphere was malleted into position with a tight fit. Trial liners were used and then removed. The prosthesis was then placed with medial impaction bone grafting from the prior humeral resection. The metaphyseal component was impacted, and the shoulder was reduced and was stable. Implants used included a Biomet® comprehensive shoulder, standard length, 11 mm, a 44 mm humeral tray with a +10 mm bearing surface, a 41 mm Glenosphere with a +3 offset, and a 28 mm standard Glenosphere base plate. Four nonlocking screws, with a 35 mm central screw, were also utilized. The procedure was performed without any complications and there was no limb length discrepancy.

### 3.2. Angiography

**Figure 1:**
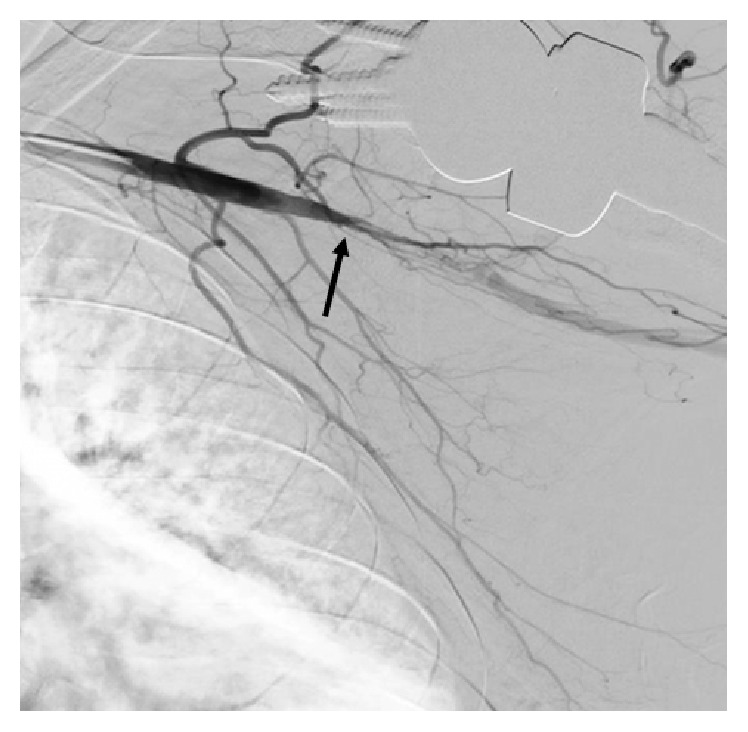
Angiography of the left upper extremity showing total occlusion of the axillary artery (arrow).

**Figure 2:**
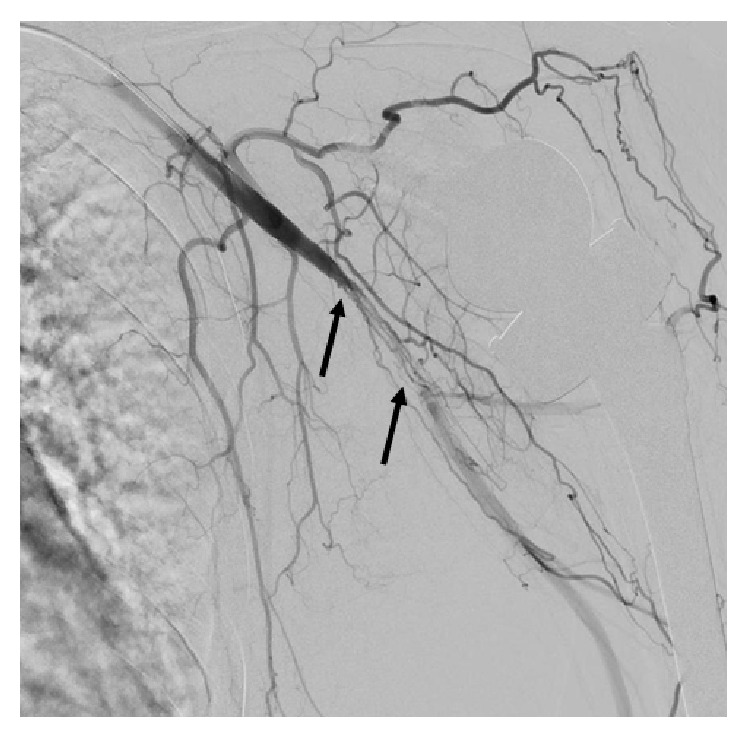
Limited angiography of the left upper extremity showing occlusion of the distal axillary artery and dissection of the proximal brachial artery (arrows).

**Figure 3:**
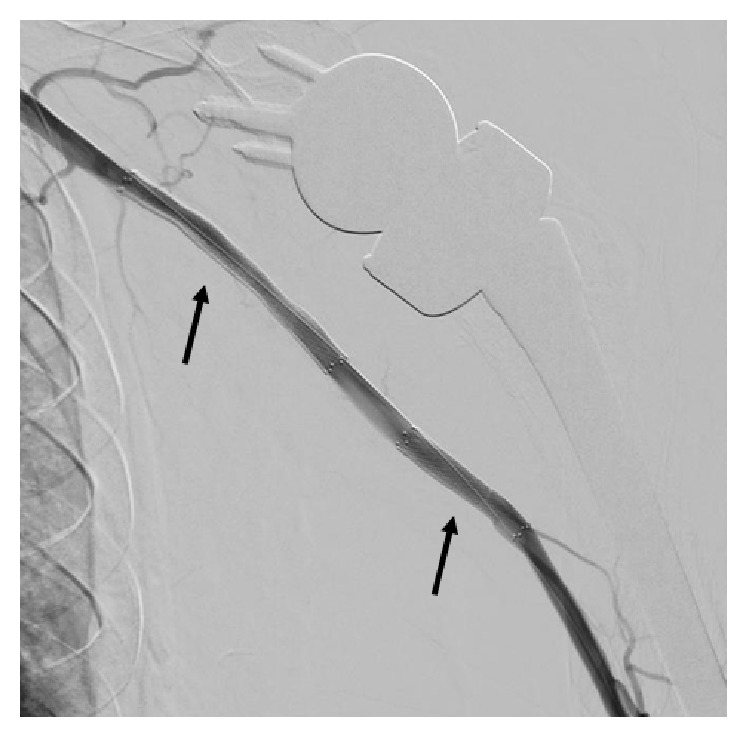
Repeat angiography of the left upper extremity after revascularization, showing a 6 × 60 mm stent in the axillary artery and a 6 × 30 mm stent in the proximal brachial artery (arrows).

Access to the right common femoral artery was obtained and selective angiography of the left upper extremity was performed. The subclavian artery was patent; however there was a 4-5 cm area of total occlusion in the axillary artery (Figure 1). For the wire to bypass the occlusion, a subintimal plane was created resulting in an area of dissection in the proximal brachial artery (Figure 2). The brachial artery distal to the dissection was patent and gave rise to patent radial and ulnar arteries. Angioplasty of the axillary artery occlusion was then performed using a 4 × 60 mm balloon. Afterwards, and to manage the dissection, a 6 × 30 mm self-expanding stent was placed in the proximal brachial artery and a 6 × 60 mm self-expanding stent was placed in the axillary artery. These stents were then postdilated with a 5 × 40 mm balloon. The repeat angiography showed excellent results with brisk flow into the forearm with no evidence of residual stenosis (Figure 3).

## 4. Outcome

The patient tolerated the procedure well and developed easily palpable brachial, radial, and ulnar pulses immediately post-op. One month after angioplasty, the patient's numbness, tingling, and the cold sensation in his hand have resolved but he continues to have pain and decreased strength. Upper extremity arterial Doppler one month after angioplasty showed significant improvement in the left digit pressures and waveforms which appeared to be essentially normal at rest. Upper extremity arterial duplex showed patent stent in the left axillary artery and brachial artery. Arterial Doppler and duplex nine months after angiography continue to show normal left digit pressures and patent axillary artery stent, respectively. He continues to take aspirin 81 mg and Clopidogrel 75 mg. He is also being treated by pain management for complex regional pain syndrome and/or local nerve injury during his shoulder repair. He may also have some residual ischemic injury to his nerves secondary to the delay in diagnosis and revascularization.

## 5. Discussion

The axillary artery and the brachial plexus originate in the posterior triangle of the neck, bound by sternocleidomastoid and trapezius muscles and the clavicle. They enter the axilla deep to the pectoralis minor muscle and run 5–20 mm medial to the anterior glenoid rim of the glenohumeral joint and therefore are susceptible during shoulder injury [1]. Although rare, vascular injury from shoulder trauma has been previously described in the literature [2, 4, 5]. Due to atherosclerosis and loss of arterial elasticity, patients over the age of 50 are seemingly more susceptible to axillary artery injury [4].

Penetrating trauma to the axillary artery is more common than blunt trauma, with blunt trauma accounting only for 6% of all injuries [2]. Nonetheless, most of the reported cases of axillary artery injury appear to be secondary to anterior shoulder dislocation [2]. Shoulder surgery can lead to axillary artery injury as well as both acute arterial thrombosis and arterial avulsion which has been described [1, 4]. This is attributable to the position of the shoulder during the arthroplasty producing torsional forces on the axillary artery similar to that seen with anterior glenohumeral dislocation [6]. Only two cases of acute axillary artery thrombosis after humeral resurfacing arthroplasty have been described in the literature [6].

Our patient was found to have axillary occlusion two months after RTSA. RTSA is a fairly new approach to shoulder replacement and its indications include previously failed anatomic or resurfacing arthroplasty as in our patient [7]. RTSA extends the humeroacromial space and lengthens the arm on average 2-3 cm. This lengthening places the axillary vessels and the brachial plexus under some longitudinal strain. Thus, vascular complications after RTSA have been reported with hematomas and phlebitis to be most common. In addition, Wingert et al. [1] have described intraoperative axillary artery avulsion during shoulder arthroplasty. According to the authors, the injury was the result of the increased tension on the neurovasculature during the procedure [1].

The delayed presentation of our patient makes us believe that the thrombosis was likely due to an intimal injury during the procedure. Gallucci et al. [3] reported a delayed axillary thrombosis two months after a nondisplaced humeral neck fracture. Gallucci et al. postulated that the initial trauma led to intimal damage. It takes an extended period of time for the subintimal dissection to occlude the artery and cause ischemia [3].

Angiography is the gold standard diagnostic study for both acute and chronic arterial thrombosis [8]. In contrast to acute arterial thrombosis which can be managed by thrombolytics, thrombectomy, end to end anastomosis, or bypass graft, chronic arterial occlusion can often be treated nonoperatively [4, 8]. If a good collateral circulation is present, medical management with pentoxifylline and aspirin can be utilized. Our patient had no radial or ulnar pulses and a cold hand suggesting suboptimal collateral flow [3] confirmed by angiogram. Conversely, we opted for operative intervention with angioplasty and stent placement.

## 6. Conclusion

Axillary artery injuries after shoulder arthroplasty are rare but may be devastating. This case stresses the importance of a high clinical suspicion and regular vascular evaluation immediately after the surgery and at a later postoperative phase. Further research is needed to better define and standardize the treatment of delayed arterial occlusive disease.
